# Joint Coupling of Awake EEG Frequency Activity and MRI Gray Matter Volumes in the Psychosis Dimension: A BSNIP Study

**DOI:** 10.3389/fpsyt.2015.00162

**Published:** 2015-11-09

**Authors:** Pauline Soh, Balaji Narayanan, Sabin Khadka, Vince D. Calhoun, Matcheri S. Keshavan, Carol A. Tamminga, John A. Sweeney, Brett A. Clementz, Godfrey D. Pearlson

**Affiliations:** ^1^Olin Neuropsychiatry Research Center, Institute of Living, Hartford, CT, USA; ^2^Department of Electrical and Computer Engineering, University of New Mexico, Albuquerque, NM, USA; ^3^The Mind Research Network, Albuquerque, NM, USA; ^4^Department of Psychiatry, Yale University School of Medicine, New Haven, CT, USA; ^5^Department of Psychiatry, Beth Israel Deaconess Medical Center, Harvard Medical School, Boston, MA, USA; ^6^Department of Psychiatry, University of Texas Southwestern Medical Center, Dallas, TX, USA; ^7^Department of Psychology, University of Georgia, Athens, GA, USA; ^8^Department of Neurobiology, Yale University School of Medicine, New Haven, CT, USA

**Keywords:** schizophrenia, schizoaffective, psychotic bipolar, joint independent component analysis, endophenotypes, biomarkers, EEG, gray matter

## Abstract

**Background:**

Many studies have examined either electroencephalogram (EEG) frequency activity or gray matter volumes (GMV) in various psychoses [including schizophrenia (SZ), schizoaffective (SZA), and psychotic bipolar disorder (PBP)]. Prior work demonstrated similar EEG and gray matter abnormalities in both SZ and PBP. Integrating EEG and GMV and jointly analyzing the combined data fully elucidates the linkage between the two and may provide better biomarker- or endophenotype-specificity for a particular illness. Joint exploratory investigations of EEG and GMV are scarce in the literature and the relationship between the two in psychosis is even less explored. We investigated a joint multivariate model to test whether the linear relationship or linkage between awake EEG (AEEG) frequency activity and GMV is abnormal across the psychosis dimension and if such effects are also present in first-degree relatives.

**Methods:**

We assessed 607 subjects comprising 264 probands [105 SZ, 72 SZA, and 87 PBP], 233 of their first degree relatives [82 SZ relatives (SZR), 71 SZA relatives (SZAR), and 80 PBP relatives (PBPR)], and 110 healthy comparison subjects (HC). All subjects underwent structural MRI (sMRI) and EEG scans. Frequency activity and voxel-based morphometric GMV were derived from EEG and sMRI data, respectively. Seven AEEG frequency and gray matter components were extracted using Joint independent component analysis (jICA). The loading coefficients (LC) were examined for group differences using analysis of covariance. Further, the LCs were correlated with psychopathology scores to identify relationship with clinical symptoms.

**Results:**

Joint ICA revealed a single component differentiating SZ from HC (*p* < 0.006), comprising increased posterior alpha activity associated with decreased volume in inferior parietal lobe, supramarginal, parahippocampal gyrus, middle frontal, inferior temporal gyri, and increased volume of uncus and culmen. No components were aberrant in either PBP or SZA or any relative group. No significant association was identified with clinical symptom measures.

**Conclusion:**

Our data suggest that a joint EEG and GMV model yielded a biomarker specific to SZ, not abnormal in PBP or SZA. Alpha activity was related to both increased and decreased volume in different cortical structures. Additionally, the joint model failed to identify endophenotypes across psychotic disorders.

## Introduction

Several independent electroencephalogram (EEG) and neuroimaging (both functional and structural) studies have been conducted in schizophrenia (SZ), schizoaffective (SZA), and psychotic bipolar disorder (PBP) (1, 2) with the aim of finding shared and specific biomarkers for these disorders. Evidence indicates considerable overlap in cognitive, structural, cortical oscillatory deficits, functional abnormalities (3, 4), and genetic sources (5, 6) across psychotic disorders, but these biomarkers have typically each been studied in isolation. Further, while most have been shown to be abnormal in relatives of affected patients, few studies have compared deficits in relatives of probands with different disorders (7, 8). A more comprehensive approach to this problem would involve studying multiple disorders and multiple biomarkers simultaneously (data-fusion) in both probands and their relatives. A data-fusion type approach combines two data modalities by capitalizing complementary and unique modality-specific information (for example, functional and structural) in an integrated model that can identify within-subject covarying patterns (linkage) across modalities (9). Such a linkage serves in identifying disease-specific biomarkers or endophenotypic markers to advance gene discovery and provide improved and disease-specific treatment (10).

For the past two decades, brain imaging and neurophysiological techniques elucidated the complex topography of neural systems and cognitive alterations in psychotic illnesses (11, 12). Structural magnetic resonance imaging (sMRI) and EEG capture spatial and temporal neural information that have characterized spatial brain network and neurophysiological abnormalities in mental diseases. Previous sMRI studies revealed gray matter volume (GMV) abnormalities especially in the frontotemporal regions of SZ patients (4, 13, 14). Eyes-closed and eyes-awake (resting-state) EEG (AEEG) studies found augmented low frequency activity in delta, theta, and alpha bands in SZ and bipolar patients (15–17). Functional correlates of oscillatory activity revealed theta oscillations to play an important role in hippocampus and prefrontal cortex (18, 19) interactions, while alpha oscillations originating in thalamocortical neuronal interactions are associated with default mode network (20) and various cortical regions (18, 21–23). One prior study reported alterations in cortical thickness related to abnormal oscillations in SZ (24); nonetheless, the biological changes accompanying the oscillatory abnormalities in psychosis are unclear and not well characterized. The data analyzed in the current study have previously been examined independently for endophenotypes and similarities in neurobiological abnormality across psychotic disorders (4, 16, 25), but the current study integrates a subset of EEG and sMRI data in multivariate analysis to examine the joint relationship between the two in psychosis.

Univariate methods that examine data from a single imaging modality based on a separate model are indeed useful in helping us to understand brain function; however, an ideal alternative is to construct a model encompassing two or more imaging modalities in a joint analysis with the potential to identify information, which is missed by separate analysis. A whole brain univariate correlation of sMRI data with EEG activity in various frequency bands is limited in sensitivity due to numerous multiple comparison procedures (>200 K voxels and multiple EEG frequencies). Despite a generally scarce literature on anatomical abnormalities associated with EEG activity in psychosis, a few prior studies have identified EEG correlates of brain volumes based on *a priori* regional hypotheses (restricted sets of brain regions) rather than whole brain analysis (26, 27). In particular, ventricular enlargement was related to aberrant alpha activity in SZ patients (27). Another study reported no association between alpha rhythm and glucose metabolic rate in occipital cortices and thalamic regions in SZ (28).

Electroencephalogram oscillatory abnormalities have been reported (29, 30) both in low frequency ranges (including delta, theta, and alpha) and in high frequency ranges (comprising beta and gamma). Increased low frequency activity was found in psychosis (31). Abnormal beta activity was demonstrated in both SZ and bipolar disorder patients and their relatives (16, 32, 33). Very few studies have examined gamma activity in psychosis, primarily due to methodological limitations in combatting high frequency noise that overlaps with gamma activity. To our knowledge, only two studies (32, 34) have examined resting-state gamma activity. One failed to find evidence for gamma abnormalities in either SZ patients or their family members (34), while the other study including SZ probands and relatives found excessive gamma activity in frontotemporal regions (32) suggesting that spectral abnormalities serve as indicators of genetic liability (endopehnotypes) for SZ. Finally, increased baseline gamma (35) (in the pre-stimulus period) in SZ was revealed during auditory steady-state stimulation. EEG alpha findings in psychosis have been inconclusive (29) with reports of increased (36–39), decreased (40, 41), and no (42, 43) alpha abnormalities in psychotic patients, perhaps due to methodological variations across these studies. A recent BSNIP EEG study displayed increased alpha in both SZ and PBP patients (16). Gray matter anomalies in the form of volumetric reduction in temporal (44–46) and parietal lobe regions (47, 48) and in cerebellum (49) have been reported in psychosis. Gray matter abnormalities in SZA disorder resembled those of SZ (4, 50). Volumetric findings in bipolar disorder have been variable with inconsistencies across several brain regions (51). A detailed review of structural MRI anomalies in SZ is presented in Ref. (52).

Within a highly networked brain, it is reasonable to expect changes in neuroelectric brain activity in one region, may be related to alterations of brain morphometry in different regions. Importantly, such a model has the potential to reveal sources of EEG alterations in brain regions that have an indirect role in the source activity, but may not be a direct generator of the EEG activity. Currently, multimodal fusion has been utilized in studies to analyze multiple data sets as it gives important decompositions and at the same time, reduces the assumptions of the data model. Examples include multimodal canonical correlation analysis (53, 54) and joint independent component analysis (jICA) (55, 56). Most multimodal studies focused on combining sMRI with fMRI (55); sMRI with evoked event-related potential (57) but to our knowledge, no report exists on integrating awake EEG (AEEG) and sMRI to examine their coupling in psychosis. The use of EEG, which allows one to measure neural activity, together with sMRI, to assess the anatomical source of activation that can provide meaningful information regarding brain structure-neural oscillation relationship and uncover underlying pathophysiology in neuropsychiatric disorders.

The integration of EEG and sMRI can be modeled by multivariate jICA, which allows one to simultaneously examine data from two or more modalities obtained from the same group of subjects by isolating the decomposed factors or source components, while permitting a shared mixing parameter (55) between the two modalities. This type of cross-information approach leverages both EEG and sMRI to enable good temporal and spatial resolution. This is especially useful for disorders like SZ and bipolar disorder where one can investigate the link between structural and functional relationships which would ultimately lead to the development of biological markers in the aspects of diagnosis and treatment efficacy (58). In the current study, we utilize biomarkers derived from AEEG and sMRI in a joint model with the primary aim of identifying the linear interaction (association based on additive linear model) between the oscillatory activity and GMV disrupted in psychosis through empirical multivariate data decomposition. Another goal is to verify whether the integrated model provides better efficacy in serving as a psychosis specific biomarker or endophenotype or as a disease-specific marker yielding better separation from healthy comparison (HC) group (compared to separate EEG and GMV data models). To our knowledge this is the first study to compare joint linear interaction between EEG and gray matter abnormalities across psychosis dimension including SZ, SZA and PBP probands, and their respective relatives. We sought to identify the symptom relationship with the integrated biomarker model by correlating with the psychopathology scores. We conducted an exploratory study using an empirical data-driven strategy to jointly evaluate EEG and whole brain sMRI data to identify both neurophysiological and concurrently occurring gray matter abnormalities in psychosis.

## Materials and Methods

### Participants

We analyzed data from 607 subjects recruited from the multisite Bipolar-Schizophrenia Network for Intermediate Phenotypes (B-SNIP) study [interested readers can refer to Ref. (2) for study details]. The participants comprised 110 HC subjects, 105 SZ probands, 87 PBP, 72 SZA probands, 82 relatives of SZ probands (SZR), 80 relatives of PBP probands (PBPR), 71 relatives of SZA probands (SZAR). The current sample (*N* = 607) was selected from all participants with both AEEG [from *N* = 1091 (16)] and sMRI (4) data, a subset of B-SNIP cohort comprising *N* = ~2441 subjects. Groups were not matched on age, gender, and ethnicity. See Table 1 for demographic information and clinical characteristics for subjects. The BSNIP study protocol was approved by the institutional review board at Hartford Hospital (Hartford), University of Texas Southwestern medical school (Dallas), University of Maryland (Baltimore), University of Chicago (Chicago), Wayne state university (Detroit), and Harvard University (Boston). All participants were explained about the BSNIP study procedure and a written informed consent approved by the institutional review board at each local site was obtained. Probands were confirmed to have a DSM-IV diagnosis of SZ, PBP, and or SZA disorder by trained and reliable clinicians using the Structured Clinical Interview for DSM-IV (SCID) Axis I Disorders. Probands were recruited from inpatient and outpatient settings at the five centers (Baltimore, Boston/Detroit, Chicago, Dallas, and Hartford) comprising the collaborative B-SNIP study via advertisements, online postings, and referral by word of mouth. Non-psychotic relatives group included in this sample comprised relatives with psychosis spectrum personality disorder (cluster A) and those with no lifetime psychiatric diagnoses (unaffected) and non-psychotic axis I (e.g., mood, anxiety) or axis II (cluster B and C disorder) diagnoses. Exclusions included presence of neurological illness, substance abuse (within 1 month), or dependence (within 6 months) or any prior extensive history of drug dependence (DSM-IV). Probands were on stable medication doses (see Table 2 for detailed medication information) for at least 4 weeks and chronic antipsychotic treatment for the duration of their illnesses, whereas HCs and relatives were free of Axis 1 psychopathology. Also, probands were assessed using the Positive and Negative Syndrome Scale [PANSS (59)], the Montgomery-Åsberg Depression Rating Scale [MADRS (60)], the Young Mania Rating Scale [YMRS (61)], and the Schizo-Bipolar Scale [SBS (1)]. Functional status was assessed with the Birchwood Functioning Scale (62).

**Table 1:** **Clinical and demographic information for the study sample**.

	HC	SZ	PBP	SZA	SZR	PBPR	SZAR	Statistic	*p* Value
Subjects (No.)	110	105	87	72	82	80	71		
Mean age^a^ (years) (SD)	38.34 (12.36)	33.03 (11.7)	35.51 (13.25)	34.47 (11.35)	44.87 (15.19)	42.66 (14.9)	44.31 (14.74)	*F*_6,600_ = 11.42	<0.001
Sex^b^ (male/female)	49/61	72/33	32/55	35/37	29/53	26/54	28/43	$\chi_{6}^{2}=35.37$	<<0.0001
Baltimore	17	31	27	16	19	26	16	–	–
Boston^c^	14	16	12	2	9	7	3	–	–
Chicago	16	14	17	9	9	11	5	–	–
Dallas	21	8	4	8	3	1	9	–	–
Detroit^c^	6	3	1	0	4	2	3	–	–
Hartford	36	33	26	37	38	33	35		–
Group-by-site effects^d^	–	–	–	–	–	–	–	$\chi_{24}^{2}=59.84$	<<0.001
Mean epochs (SD)	111.26 (35.12)	115.96 (35.35)	113.62 (37.93)	111.07 (34.57)	107.48 (30.71)	118.06 (38.3)	110.39 (34.41)	*F*_6,600_ = 0.87	0.52
Ethnicity^e^								$\chi_{24}^{2}=65.97$	<<0.0001
Caucasian	50	46	59	36	48	60	50		
African-American	37	47	13	18	22	9	15		
Hispanic	11	5	8	12	8	8	3		
Asian	5	0	2	0	2	0	1		
Mixed	7	7	5	6	2	3	2		
CPZ^f^ equivalents		484.9 (386.78)	307.9 (269.34)	465.38 (357.7)	203.7 (188.82)	72.9	346.29 (278.2)		
PANSS-positive									
Range		7–31	7–23	7–32	–	–	–		
Mean (SD)		16.34 (5.46)	11.76 (4.1)	17.24 (5.38)	–	–	–		
PANSS-negative									
Range		7–33	7–30	7–30	–	–	–		
Mean (SD)		15.4 (5.53)	11.67 (4.06)	15.22 (5.04)	–	–	–		
PANSS-general									
Mean (SD)		30.64 (8.44)	26.98 (7.26)	33.29 (8.6)	–	–	–		
SBS									
Mean (SD)		7.88 (1.1)	1.47 (1.29)	5.11 (1.44)	–	–	–		
YMRS score									
Mean (SD)		5.66 (5.39)	5.13 (5.9)	8.3 (6.68)	–	–	–		
MADRS score									
Mean (SD)		8.88 (8.08)	10.04 (9.79)	14.94 (10.12)	–	–	–		

*^a^SZ probands <HC group*.

*^b^Disproportionate number of males in probands and disproportionate number of females in psychotic bipolar probands*.

*^c^Since identical EEG equipment was used to collect data at both Boston and Detroit sites, which were managed by the same investigator (two sites were merged in the study). Site was used as a factor with five levels in the statistical analysis*.

*^d^Patients, relatives, and healthy controls were not uniformly distributed across data collection site*.

*^e^Race was not balanced across groups, particularly there were more Caucasians*.

*^f^CPZ was available for only one relative of PBP*.

*CPZ, chlorpromazine; HC, healthy comparison; PANSS, Positive and Negative Syndrome Scale; PBP, psychotic bipolar disorder; PBPR, relatives of psychotic bipolar probands; SBS, Schizo-Bipolar Scale; SZ, schizophrenia; SZA, schizoaffective; SZAR, relatives of schizoaffective probands; SZR, relatives of schizophrenia probands*.

**Table 2:** **Talairach coordinates for brain regions associated with alpha activity derived from joint independent component analysis**.

Regions	Brodmann	*L* random effects: Max *Z* (*x*, *y*, *z*)	*R* random effects: Max *Z* (*x*, *y*, *z*)
**Positive**			
Culmen	*	4.1 (0, −50, −10)	3.6 (3, −50, −8)
Uncus	28	–	3.7 (19, −6, −25)
**Negative**			
Parahippocampal gyrus	28, 34	3.26 (−18, −10, −18)	4.2 (40, −38, 36)
Supramarginal gyrus	40	3.1 (−36, 17, 21)	
Inferior frontal gyrus	*	3.99 (−33, 34, −16)	3.1 (33, 33, 3)
Middle frontal gyrus	*	3.5 (−36, 26, 29)	3.3 (42, 18, 38)
Superior frontal gyrus	*	3.4 (−27, 46, 17)	–
Inferior parietal lobule	40	–	3.6 (43, −37, 39)
Inferior temporal gyrus	20	3.6 (−56, −26, −20)	–

*The regions were identified in the gray matter component using a threshold of |*Z*| ≥ 3*.

*denotes Brodmann region not defined.

### EEG Data Acquisition and Processing

**Figure 1:**
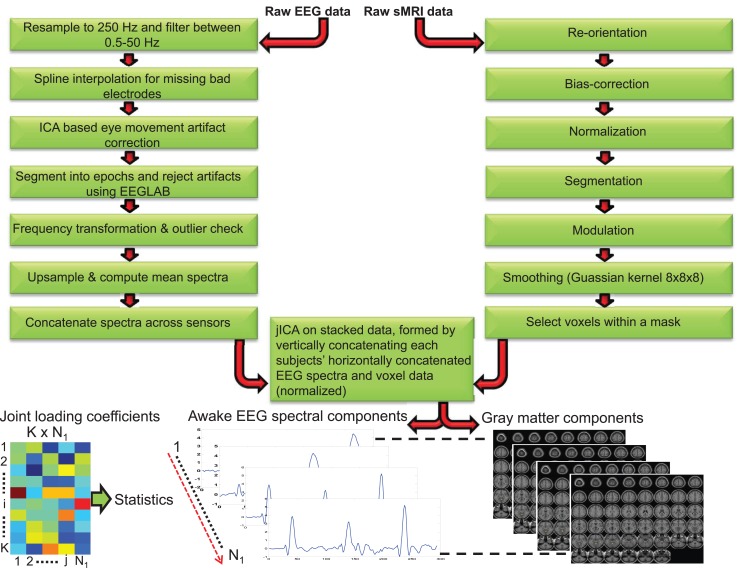
**Schematic depicting the processing steps for awake electroencephalogram and gray matter data**. The processed EEG spectra (concatenated spatially) and voxels data (both data normalized) were concatenated for each subject and Joint Independent component analysis (jICA) was applied on the stacked data (vertically concatenated across all *K* = 607 subjects). *N*_1_ = 7 components were derived using jICA. The component whose loading coefficient revealed between-group differences was segmented to yield the EEG frequency and linearly associated gray matter component. The dotted line between the components indicates linearly paired EEG frequency and gray matter sources obtained from jICA.

EEG data were acquired with an electrode cap fitted with 66 electrodes, conforming to scalp locations according to the International 10–10 system. Instructions were given to participants to sit relaxed in a straight-backed chair with eyes open and remain still while looking at a fixation cross on a monitor. Identical EEG equipment (Neuroscan Compumedics, Charlotte, NC, USA) standardized according to the same guidelines was used at all the sites. All electrodes were adjusted for impedance ≤5 kΩ. Electrooculography (EOG) was used to monitor eye movement horizontally with electrodes next to both eyes, and vertically with electrodes above and below the right eye. EEG signals were digitized at the rate of 1000 Hz with 0.5 Hz low-frequency and 100 Hz high-frequency filters. Raw EEG data were visually inspected to identify bad sensors and were fixed by applying a spherical spline interpolation (very few subjects’ data were interpolated such that each had no ≥8% of bad electrodes); refer to Ref. (16) for the data processing details and see Figure 1. Blink artifacts were corrected using independent component analysis (63). EEG data were also denoised for EKG/EMG artifacts using ICA based artifact cleaning methods (64–67). The components were screened both visually and using kurtosis and spectral deviation metrics for identifying and removing sources of EKG/EMG artifacts. Individual EEG trials/epochs were generated by segmenting the continuous data into 50% overlapping segments of 2.048 s, followed by baseline correction to the mean value of the entire trial. Trials containing extreme outliers (exceeding a 150 uV threshold), improbable distribution (≥3.25 SD from mean), or kurtosis behavior (3.75 SD from mean) or abnormal spectra were discarded. Further pruning was done by frequency-transforming data using a Hamming window and subsequently rejecting epochs that were (±) 4 SD from the mean spectral amplitude at all frequency points between 0.5 and 50 Hz. Finally, EEG data were visually inspected by trained research personnel to ensure valid brain EEG patterns were retained for subsequent analyses.

### Structural Magnetic Resonance Imaging Acquisition and Voxel-Based Morphometry Procedures

All MRI images were collected with 3-T scanners (GE Signa, Philips Achieva, Siemens Allegra, and Siemens Trio) as described elsewhere (4). High-resolution isotropic T1-weighted MP-RAGE sequences (TR = 6.7 ms, TE = 3.1 ms, 8° flip angle, 256 × 240 matrix size, total scan duration = 10:52.6 min, 170 sagittal slices, 1 mm slice thickness, 1 mm × 1 mm × 1.2 mm voxel resolution) were acquired according to the Alzheimer’s Disease Neuroimaging Initiative (ADNI) protocol.^1^ Foam pads and ear plugs were given to minimize head motion and scanner noise.

### VBM

Structural MR images were processed using the VBM8 toolbox^2^ as implemented in SPM8 with default settings (see Figure 1). The following steps were conducted for the T1-weighted MP-RAGE images: reorientation, bias-correction, tissue classification, spatial normalization to Montral Neurological Image (MNI) space followed by high-dimensional non-linear diffeomorphic anatomical registration through exponentiated lie algebra (DARTEL) normalization that incorporates correction for individual brain size (68). The optimally processed and normalized gray matter segments were modulated by the amount of warping to maintain the total GMV (69) and finally smoothed with an 8 mm × 8 mm × 8 mm isotropic Gaussian kernel. Voxels within a predefined gray matter mask (absolute threshold = 0.3) were selected and used for the subsequent jICA.

### Joint Independent Component Analysis

Typical univariate studies quantify the EEG power and volumes in several *a priori* defined frequency bands and brain regions of interest respectively, and evaluate the correlations between the two. This approach is limited by the numerous statistical corrections across several frequency bands, numerous EEG leads and brain regions of interest to eliminate false positives. In this study, we analyzed the whole EEG oscillation spectrum spanning (between 1.5 and 50 Hz) from low to high frequency in conjunction with whole-brain gray matter data. The jICA decomposes the data into components that are essentially spectrally filtered EEG data covarying with regional volumes (i.e., each component represents a spectral activity within a frequency band and linearly related regional brain volumes). The EEG and sMRI data were analyzed by applying jICA (9, 55) in the fusion ICA Toolbox.^3^ jICA is a multivariate statistical approach that extracts hidden factors by exploiting the co-variation across two data modalities with high dimensionality, while simultaneously maximizing the statistical independence in the factors. The loading coefficient (LC) of the hidden factors captures the linear linkage between the modalities. The linkage parameter and associated hidden source components are derived from the data empirically (data-driven). One advantage of the multivariate approach is the number of statistical corrections is limited to the few hidden factors as opposed to correcting for all the voxels (>200,000) in a univariate model. In simple terms *x* = AS, where the joint independent sources (S) are linearly mixed by a common mixing (loading) parameter (A) to achieve the data matrix *x* = [*x*^EEG^, *x*^smri^]. To eliminate bias in the joint analysis resulting from the high dimension of the GMV data (comprises approximately >200 K voxels), a balanced representation of the data from both modalities was achieved by interpolating (upsampling) the EEG frequency activity for each trial and averaging the trials to yield the mean frequency amplitude for each channel (3178 frequency points/channel between 1.5 and 50 Hz). The multichannel EEG data was spatially concatenated to form a single vector of (1 × 203392 = 64 × 3167), which in turn was transformed to subject × frequency data matrix (*x*^EEG^ = 607 × 203392). Similarly, the GMV data of each subject was converted to a single spatial vector (1 × 202145 voxels) and subsequently transformed to form a subject × spatial GMV data matrix (*x*^smri^ = 607 × 202145). The EEG and GMV data matrices were normalized by respective scaling factors such that the sums of squares were matched between the two modalities, while maintaining the relative amplitude of the frequency and GMV data. The final data matrix for jICA was obtained by concatenating the EEG and GMV (see Figure 1) data matrices [*x* = (*x*^EEG^, *x*^smri^)].

It is not straightforward to estimate the model order for the combined EEG and GMV data; hence we adopted a strategy employed in prior jICA study (70). An initial estimate of the number of independent components was determined using minimum description length (MDL) criteria (71) for EEG and GMV data separately. Model order is an estimate (approximate) of the low order components that represent the patterns contributing to the data. For ICA based studies model order is computed based on stability criterion (72). ICA is repeatedly applied on the data with random initializations and the resulting components are grouped into clusters that are assessed by computing a stability index [ICASSO (72, 73)]. Using the initial MDL computed model order as reference, multiple runs of iICA was evaluated to identify stable EEG frequency and gray matter components. With a high model order (>10), the EEG frequency components disintegrated around the dominant modes. The final model order was selected as 7 (with stability index ranging between 0.91 and 0.97) and iICA was evaluated using infomax ICA (74). Those components for which the LC yielded a significant group main effect were examined further. The LC captures the linear aggregate relationship between the EEG frequency activity and GMV measures derived from the two modalities. A higher component LC for a subject signifies increased contribution to the component. The constituents of the EEG frequency and gray matter component were identified by converting the component to *Z*-scores and applying a threshold |*Z*| ≥ 3. The robustness of the component was tested by evaluating iICA in leave one subject out sampling procedure (*N* = 607 runs) and computing the Pearson correlation between the component obtained in each run and the component extracted from the original data.

### Statistical Data Analysis

#### Joint ICA Analysis

jICA identified 7 EEG–GMV joint components, whose loading or mixing parameters were statistically assessed for group difference. First, all probands (SZ + SZA + PBP) were clustered as a group and compared against HC, followed up with a one-way analysis of covariance (SPSS v17.0 Statistical Package for the Social Science, Chicago, IL, USA) with three between subject factors (group: SZ, SZA, PBP, SZR, SZAR, PBPR and HC; sex: male/female; data collection site: five levels). Age and number of EEG trials were used as covariates. The *p*-values for the components were adjusted to correct for multiple comparisons (*p* < 0.05/7). Significant main effect was followed up with *post hoc* tests to assess pair-wise group comparisons adjusted using Bonferroni correction. Separate regression (accounting for age, sex, site, and number of EEG trials) was carried out to examine the associations between the loading parameters of the significant component and clinical variables including PANSS, MADRS, YMRS, SBS scores, and chlorpromazine equivalents (75) in probands. Additionally, medication effect on loading parameters were examined using multiple regression by taking into account simultaneous medication usage, modeled as separate dichotomous discrete variable for use or no use of antipsychotics, antidepressants, and mood stabilizers. This analysis was restricted to only those probands in which the loading parameters characterized abnormality.

## Results

### Joint EEG–GMV Components

**Figure 2:**
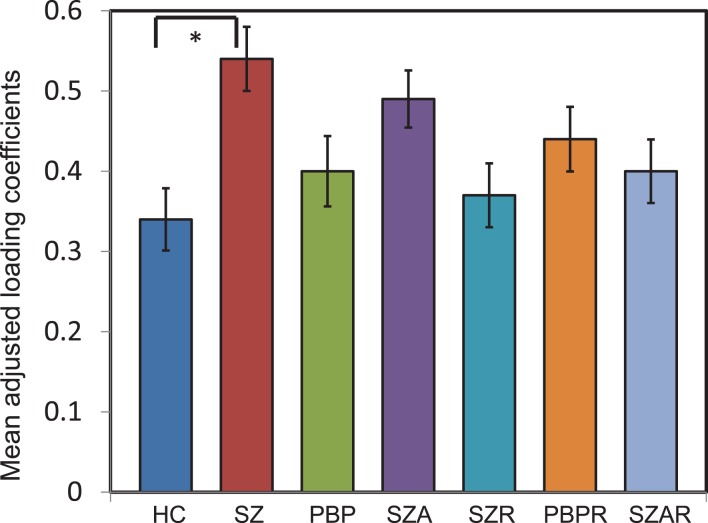
**Mean loading coefficient for the joint electrophysiology-gray matter component from joint independent component analysis**. The loading coefficients are plotted for joint component IC5. Analysis of covariance identified IC5 to show significant group main effect. *Post hoc* tests with Bonferrroni correction identified the difference was prominent between schizophrenia (SZ) probands and healthy comparison (HC) subjects. No other proband groups or relatives of probands differed. *indicates significance at the *p* < 0.05 level. The loading coefficients were adjusted for discrete factors (sex, site) and covariates (age and number of epochs). Error bars represent SD.

**Figure 3:**
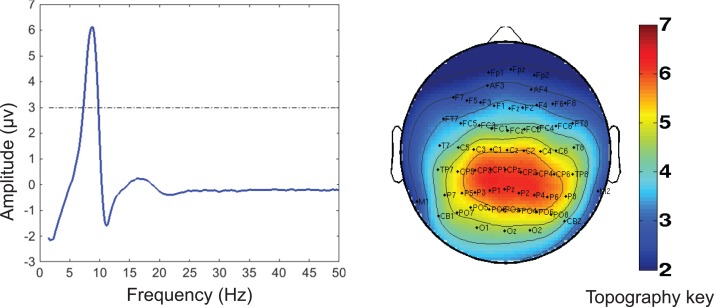
**Electrophysiology (EEG) portion of the joint component (IC5) obtained from the multivariate joint analysis**. Left: the frequency waveform of the component was plotted at the channel location at which the component was peaking. The dotted line indicates the threshold level of |*Z*| = 3 for identifying the constituent of the frequency component. The peak amplitude was within the slow alpha frequency range (7.5–10 Hz). Right: the topography associated with the alpha component indicates a posterior distribution. The topography was plotted by mapping the peak in the alpha range for each channel in the EEG portion of the joint component. The component was converted to *Z*-scores.

**Figure 4:**
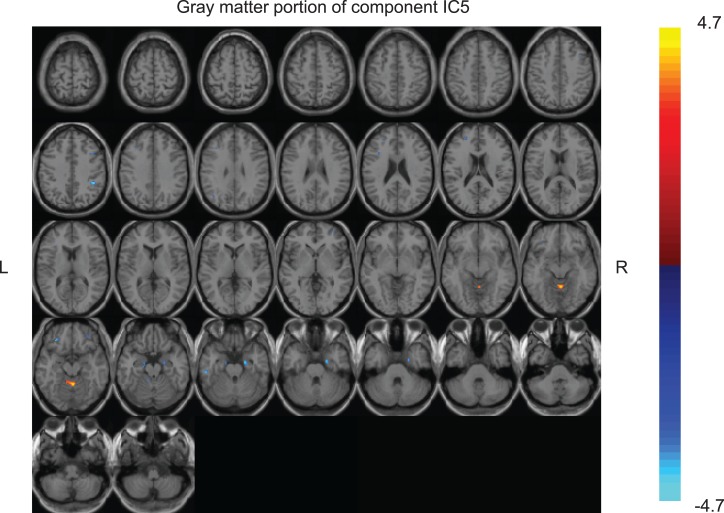
**Gray matter portion of the joint component IC5 obtained from the multivariate joint analysis**. The gray matter component was converted to *Z*-scores and the individual brain regions comprising the component were identified with a threshold of |*z*| ≥ 3 (regions with at least 20 voxels).

Of the seven components derived from these data by jICA (IC1-7 explaining ~88% of variance), only one component’s loading (IC5 with ~16% explained variance) showed significant main effect (*F*_1,365_ = 9.96, *p* < 0.0017) in the initial analysis contrasting psychosis (SZ + SZA + PBP) vs. HC. IC5 also showed significant group (conventional DSM group comparisons) main effect, (*F*_6,593_ = *2.99*, *p* < 0.006), indicating that the magnitude of linear linkage between EEG and GMV in that component was significantly different across DSM groups. *Post hoc* tests revealed that the loading for IC5 was significantly higher in SZ probands compared to HC (*p* < 0.004 after Bonferroni correction for multiple group comparisons; see Figure 2), reflecting that strength of the coupling between EEG and GMV was increased in SZ compared to HC. No other proband groups or their relatives showed difference vs. HC. The dominant EEG activity and gray matter regions representing the component was identified by the applying a threshold on the *Z*-scored component (similar to identifying outliers). The EEG feature comprising component IC5 was selected with a threshold |*Z*| ≥ 3 (70, 76) [outliers that are 3 SD (*p* < 0.01) away from the mean], yielding slow alpha activity between 7.4 and 9.6 Hz and maximal (at Pz) in the posterior channels (Figure 3). The EEG frequency component is the waveform at the channel location (Pz) where the component was peaking. The associated topography was obtained by mapping the peak of each channel comprising IC5 in the alpha frequency range (7.5–10 Hz). The individual gray matter regions (Figure 4) contributing to IC5 are listed in Table 3 with their Talairach coordinates and *Z*-scores. Increased posterior alpha was associated with lesser volume in parahippocampal gyrus, inferior temporal gyrus, left middle frontal gyrus, right inferior parietal lobule, and greater volume in culmen and uncus. Although the threshold used in this study is arbitrary, a high threshold results in fewer regions (small sized) associated with oscillatory activity, while a low threshold leads to false positives. The minimum cluster size for the gray matter region was chosen as 20 voxels; however, several of the identified regions comprised 53–482 voxels. Thus, we exercise caution on the findings as these are sensitive to several parameters including the threshold and voxel size. The component IC5 was robust with a stability index >0.9 from the leave-one-out analysis.

**Table 3:** **Medication information for study sample**.

	SZP (*n* **=** 105)		PBP (*n* **=** 87)		SZAP (*n* **=** 72)		SZR (*n* **=** 82)		PBPR (*n* **=** 80)		SZAR (*n* **=** 71)		HC (*n* **=** 110)	
	*N*	%	*N*	%	*N*	%	*N*	%	*N*	%	*N*	%	*N*	%
No medication taken	5	4.8	5	5.7	3	4.2	66	80.5	53	66.3	53	74.6	107	97.3
Not on psychotropic medications	8	7.6	23	26.4	10	13.9	80	97.6	78	97.5	2	2.8	0	0
On more than one psychotropic medications	12	11.4	2	2.3	5	6.9	0	0	0	0	0	0	0	0
Anticholinergic/antiparkinsonian	19	18.1	6	6.9	9	12.5	0	0	0	0	0	0	0	0
Antidepressant (any)	38	36.2	39	44.8	42	58.3	8	9.8	23	28.8	12	16.9	3	2.7
A. Tricyclic	0	0	3	3.4	3	4.2	0	0	2		2		0	0
B. MAO inhibitors	0	0	0	0	0	0	0	0	0	0	0	0	0	0
C. SSRI/SNRI	29	27.6	22	25.3	30	41.6	7	8.5	18	22.5	9	12.7	2	1.8
D. Miscellaneous	15	14.3	23	26.4	22	30.6	1	1.2	5	6.3	2	2.8	2	1.8
Antipsychotic (any)	97	92.4	64	73.6	63	87.5	2	2.4	2	2.5	2	2.8	0	0
A. First generation	21	0.2	5	5.7	12	16.7	0	0	0	0	0	0	0	0
B. Second generation	88	83.8	61	70.1	55	76.4	2	2.4	2	2.5	0	0	0	0
Anxiolytic/hypnotic	21	0.2	22	25.3	25	34.7	9	11	7	8.8	5	7	1	0.9
Mood stabilizer (any)	22	21	63	72.4	44	61.1	0	0	3	3.8	5	7	0	0
A. Lithium	7	6.7	25	28.7	12	16.7	0	0	1	1.3	0	0	0	0
B. Anticonvulsants	17	16.2	48	55.2	35	48.6	0	0	2	2.5	5	7	0	0
Miscellaneous, centrally active	2	1.9	5	5.7	5	6.9	0	0	0	0	1	1.4	0	0
Stimulants	3	2.9	10	11.4	2	2.8	1	1.2	2	2.5	2	2.8	0	0

*HC, healthy comparison; PBP, psychotic bipolar disorder; PBPR, relatives of psychotic bipolar probands; SZ, schizophrenia; SZA, schizoaffective; SZAR, relatives of schizoaffective probands; SZR, relatives of schizophrenia probands*.

**Figure 5:**
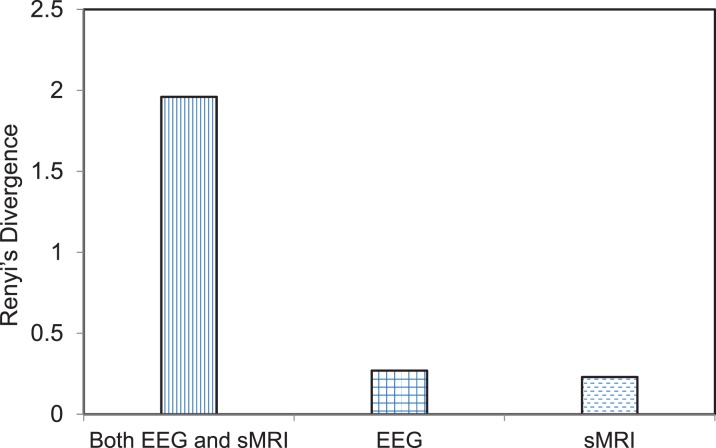
**Bar graph depicting the divergence score on the individual and combined feature space**. The feature space includes joint electrophysiology (EEG) frequency activity and gray matter volumes (GMV) and either of the two separately. Renyi’s divergence compares the distribution of the back-reconstructed component between schizophrenia probands and healthy comparison groups. The divergence is calculated for the component that best separated the two groups for the joint modality (EEG + sMRI) as well for each modality. The *p* values associated with the divergence measure for the joint modality as well as the individual EEG and GMV data were *p* = 0.11, 0.36, and 0.45, respectively. The statistic associated with the divergence was evaluated by re-running (*N* = 500) the back-reconstruction with shuffled group labels and evaluating the ratio of the number of times the divergence measure exceeded the original value to the total number of runs.

To evaluate the sensitivity of the jICA approach compared to mass univariate approach, we conducted simple regression between the maximal gray matter voxel and the peak amplitude of the EEG frequency component. The maximal voxel was located in supramarginal gyrus. The peak EEG frequency amplitude was at channel Pz at 8.6 Hz (within alpha band). The GMV and EEG frequency activity in the original subjects’ data at these two maximal locations was correlated (*r* = −0.16, *p* = 0.000064) in the univariate analysis. This was the maximum correlation and the associated significance clearly does not overcome the threshold for multiple comparisons across 200 K voxels. Separate ICA was applied on EEG and GMV data and the component that best differentiated SZ probands from HC in each modality was back-reconstructed using the LCs and original data for each group including SZ and HC. The number of independent components for EEG and GMV data was 6 and 12, respectively. The best differentiating (SZ vs. HC) EEG component comprised theta activity (5.5–8.3 Hz), maximal at Cpz, while the best gray matter component comprised several regions including vermis, inferior parietal lobule, thalamus, and parts of frontal lobe including left and right inferior frontal gyrus. The back-reconstruction procedure was also applied to IC5 derived from the joint analysis and the distribution of the back-reconstructed component was compared between SZ and HC for the joint modality as well as for each modality independently using Renyi’s information-theoretic divergence (77) criteria. Renyi’s divergence is an inferential statistical distance metric that quantifies the difference between the distributions of two groups or classes (78). The divergence is evaluated using a non-parametric estimator. Greater divergence indicates a larger distance between the distribution of SZ and HC group back-reconstructed component. The statistic associated with Renyi’s divergence was evaluated by re-running (*N* = 500 runs) the group back-reconstruction procedure with shuffled group labels and estimating the divergence for each run. The *p* value was computed as the ratio of the number of times the divergence exceeded the original value to the total number of runs. Thus, the *p* values for the joint model as well as the individual EEG and GMV model were *p* = 0.11, 0.36, and 0.47, respectively. Although none of the divergence measures were significant, the joint model displayed a trend. The joint model revealed a greater divergence (see Figure 5), or separation between the distribution of the component (IC5) for SZ vs. HC in comparison to the distribution contrast for the separate ICA models of EEG and GMV, indicating that the SZ abnormality is better differentiated by combing both EEG and GMV data.

No significant correlations were found between the loading parameters and PANSS, MADRS, YMRS, SBS scores and chlorpromazine equivalent dose. Further no association between the loading parameters and current use of antipsychotics, antidepressants and mood stabilizers were found among SZ probands. All the associations were non-significant at *p* = 0.05.

## Discussion

Numerous brain regions are compromised in the form of functional and structural abnormalities in psychosis (79–81). Prior studies have identified regions of gray matter abnormalities including the thalamus, cerebellum, temporal lobe and lateral frontal regions (4, 52, 82). EEG studies revealed low frequency delta, theta, and alpha abnormalities in SZ and PBP probands (16, 27, 29, 31, 38). The co-occurring abnormalities in both EEG and sMRI in psychosis are unknown and poorly defined. Multiple endophenotypes and/or biomarkers have been identified in psychosis, but it is not clear whether these are related to each other or independent; determining this might help clarify whether similar or separate biological mechanisms underlie the disorder. As a first step, we conducted an exploratory whole-brain multivariate jICA analysis to demonstrate the biological concomitants of oscillatory activity by integrating AEEG frequency activity and GMV from sMRI data across subjects in the B-SNIP study and examining their linear relationship that models the shared between-modality relationships in psychosis. It should be emphasized that join analysis (joint data fusion) only captures components that exhibit a shared relationship between the two modalities.

### Joint Analysis Findings

Brain rhythms encompass wide range of putative functions including feature binding and information transfer between local and long range brain circuits (83). Similarly, gray matter brain regions and circuits serve multiple functions (e.g., cognitive and sensory processes) based on information processing governed by neurons, which are also primarily responsible for generating brain oscillations. Therefore, it is reasonable to expect some relationship between EEG oscillations and variations in brain structure. Moreover, generation and regulation of alpha oscillations can be linked to brain regions that are direct source generators, such as visual occipital cortex (84) or regions that are not direct source generators but interact with actual sources. The neurobiological mechanisms of brain oscillations are complex. We briefly discuss the plausible mechanisms underlying the generation of alpha oscillations. Alpha rhythms were initially observed in the occipital cortex (85); however, they have also been noted in somatosensory cortex (86), auditory cortex (87, 88), and the prefrontal cortex (89). There remains a considerable debate regarding the neural basis of alpha activity; however, studies have demonstrated that alpha oscillations are generated in neocortex, thalmo-cortical networks (90–92) as well as in hippocampus and reticular formation (84), presumably due to reciprocal inhibitory and excitatory neuronal interactions regulated by gap junctions in inhibitory interneurons (93). Generation of awake alpha is associated with the bursting of specialized class of high-threshold thamocortical cells at depolarized membrane potentials (92). Recent evidence implicates muscarinic aceylcholine receptor or metabotropic glutamate receptor agonist on thalamic reticular and thalamocortical cells during alpha generation (92).

The present study identified slow alpha rhythm including the upper end of the theta frequency, whose origin has been associated with hippocampal spiking (94) and activation (95, 96), linked to memory formation. Prior MRI studies have demonstrated functional relationship between dorsal attention network, posterior network involving temporal-occipital regions and default mode network comprising superior, medial frontal gyrus and inferior parietal lobule (21) with alpha rhythm. Alpha activity was also correlated with activations in middle and inferior frontal gyri (22, 96–98), inferior parietal lobule (99), middle temporal gyrus, and cerebellum (20). Source localization analysis of alpha points to the role of posterior occipital lobe (100–102) where the visual cortical regions are located, extending to subcortical structures involving insula, cuneus, and precuneus (100, 103). Glutamate and GABAergic (excitatory and inhibitory) neurotransmitters play an important role in abnormal brain oscillations in SZ (104). Additionally, GMV loss in SZ can be partly attributed to reduced neuropil (105, 106), plausibly resulting from NMDA hypofunction that reduces the excitation of inhibitory GABAergic interneurons leading to disinhibition of glutamatergic pyramidal neurons (107). Thus, it is critical to study the relationship between alpha activity and structural alterations to understand the disease pathology, which is feasible using joint analytic techniques. The current findings implicate several brain regions directly or indirectly associated (both positively and negatively, i.e., anti-correlated) with alpha oscillations. It is plausible that these regions are connected via white matter tracts and that this plays a joint role in their association with alpha oscillations. For example, alpha oscillations generated in the primary visual cortex are coordinated and distributed (108) to other regions including hippocampus, precuneus, inferior frontal, lingual and parahippocampus gyri, as these regions are connected via white matter pathways. Moreover, the white matter tracts (in fronto-temporo-occipital lobes) (109) assessed using fractional anisotropy (FA) is disturbed in SZ (110–112) and such disturbances have functional correlates (113). Abnormalities detected using diffusion tensor imaging based FA measures are indicative of disturbed organization/direction of white matter fibers or connections between brain regions.

The novel joint analysis reported in this study identified a single component, IC5 comprising alpha activity (with posterior topography) and gray matter regions including parahippocampal gyrus, left inferior temporal gyrus, left middle frontal gyrus, right supramarginal gyrus, and right inferior parietal lobule. This component was abnormal and specific to SZ probands, contrary to our hypothesis that EEG–GMV linkage is psychosis-specific. Also, no first-degree relatives in any proband groups exhibited abnormalities compared to HC. Prior studies documented both EEG (16) and GMV (4) abnormalities in relatives with other non-psychotic axis 1 disorders (mood and anxiety) and psychosis spectrum personality disorder (axis II), however in the current sample the size of cluster A subjects was too small (*N* = 8) to compare with probands and HC. The current findings indicate that the joint model including EEG and sMRI capture and characterize a SZ-specific abnormality. Additionally, the specificity in differentiating SZ from HC is higher (bot not significant) compared to independent investigations of these data. Prior studies have shown certain degree of overlap in oscillatory and morphometric abnormalities in a direct contrast of both SZ and PBP (4, 16, 25), perhaps reflecting similar biological disturbances in both disorders, however our preliminary findings suggest that the linear relationship between alpha oscillations and co-occurring volumetric changes are unique to SZ and could be used in future investigations for elucidating the disease pathology.

### Alpha Oscillations

Numerous EEG studies have shown SZ patients exhibiting abnormal resting neural oscillations across the entire spectrum including delta, theta, alpha, beta, and gamma frequency ranges (31, 32, 38, 114). SZ probands in the present study revealed an augmented alpha oscillatory activity, emphasized over the posterior region. Functional correlates of alpha include inhibition (115), default mode (21, 116), arousal (117), attention, and memory processes (84, 118). Prior studies have reported conflicting findings on the direction of alpha activity (increases or decreases) in SZ probands, perhaps due to the normalization of frequency band powers and differences in methodology and sample characteristics such as age and psychotropic medications. Our data suggest that morphometric changes in the frontal, parietal, and temporal regions may subserve directly or indirectly as a biological substrate for altered EEG alpha activity. There are mixed reports of both positive and negative occipital lobe findings in SZ, probably due to different methodology and regions of interest definitions that could account for these inconsistencies. Another possible explanation is occipital abnormalities in SZ may be influenced by sex, although the present study did not find diagnostic group-by-sex interactions. Previous studies have reported few of the brain regions identified in this study as specific sources of EEG alpha activity (96, 100, 119). Further, the current findings are partially supported by previous EEG–fMRI studies that showed bold activation in middle frontal, inferior temporal gyrus, inferior parietal lobule and hippocampus, associated with EEG alpha rhythm (20, 21, 23, 96, 98, 99). The direction of the alpha-GMV relationship identified in this study is consistent with the inverse relationship between alpha and bold activation reported in Ref. (23).

### sMRI Regions

Increased posterior alpha was associated with gray matter reduction in the left inferior temporal gyrus, left middle frontal gyrus, right supramarginal gyrus, right inferior parietal lobule and parahippocampal gyrus, brain regions previously implicated in the pathophysiology of SZ (11, 120–122). The inferior parietal lobule, which is a major component of the heteromodal association cortex (123), is crucial in sustaining attention to sensory modalities and thus, SZ probands may exhibit impairment of sensory integration to a certain degree (123, 124). Similarly, the supramarginal gyrus is efficient at integrating incoming sensory data and several studies have showed volume reduction in SZ (48, 125, 126). The middle frontal gyrus considered to be part of the dorsolateral prefrontal cortex, plays an important role in working memory known to be compromised in SZ patients (122). Additionally, a meta-analysis of 31 voxel-based morphometric studies showed reductions of GMV in the left middle frontal gyrus (127). Alpha was also associated with larger GMV in culmen and uncus regions. This finding is contrary to majority of prior reports, except for one study noting greater vermis volume in SZ (128). This result indicates that direction of volume changes is different in certain regions as opposed to the diffuse reductions noted in SZ. The primary regions related to alpha in this study were mainly associated with sensory and attention process, similar to the known functional correlates of alpha, suggesting that sensory integration and related processes are disturbed in SZ, a hallmark of the disease (129, 130).

### Medication Effects

Prior reports support the influence of medications, in particular antipsychotics on both GMV (131, 132) and EEG oscillatory activity (133). Our study did not identify a significant relationship between the EEG–GMV linkage and chlorpromazine equivalents. We also tested for simultaneous medication use (modeled as binary variable, indicating current use/not using antipsychotic, antidepressants or mood stabilizers) effects on the linear EEG–GMV relationship and observed no significant impact. We exercise caution on these negative results, as our study probands were prescribed multiple medications with varying durations and dosages. It is difficult to separate disease from medication effects, and this cannot be achieved by only examining probands. Although relatives were included in this study, no joint abnormality was observed in them; thus our ability to rule out medication induced effects is less definitive.

### Advantages and Limitations

The strengths of our study encompassed the relatively large sample size and the ability to utilize the full array of the EEG sensors and entire frequency spectrum (1.5–50 Hz) along with high-dimensional sMRI data. The jICA allows one to investigate the relationship between EEG and GMV in statistically efficient way by decomposing the components on the feature space, and examining their shared dependence in patients, compared to the mass univariate analysis. The jICA approach assumes a common distribution for both EEG and sMRI joint sources across all subjects but with variations in their linear relationship. Limitations include unbalanced groups on demographic variables including age and sex, whose effects were controlled in the statistical model. Despite identifying a SZ-specific biomarker, the data-driven approach in the current study failed to reveal dimensional relationship with psychosis symptom scores, as study patients had varying illness chronicity, were in stable phases of illness (restricting range of symptoms) and were taking numerous medications that may affect symptom severity. An alternative approach would be to examine first-episode patients with symptoms independent of medication influence, although such patients are both rare and generally difficult to recruit. The study did not collect detailed longitudinal medication histories for probands to completely assess possible historical medication influence on the biomarkers. We emphasize caution in interpreting the current findings, since these were dependent on several parameters including threshold and voxel cluster size, which were used in identifying the regions and spectral features that were linearly linked in the joint ICA component. We were unable to validate the findings reported in the present study due to lack of an independent replication sample; however, the findings were in agreement with those reported in several prior studies demonstrating similar regions associated with alpha rhythm.

## Conclusion

We conducted an exploratory study using a novel multivariate joint analysis for evaluating the linear relationship between AEEG frequency activity (between 1.5 and 50 Hz) and their biological concomitants in the form of morphometric changes in psychosis. Contrary to our initial prediction that linked abnormalities will be seen in psychosis (present in all three SZ, SZA, PBP proband groups), we identified a single EEG–GMV component comprising abnormal alpha activity accompanied by structural abnormalities in several brain regions; the abnormal EEG–GMV linear relationship was specific only to SZ probands. These ­findings suggest that EEG–GMV linkage serve as a potential disease-specific biomarker for SZ, which may be useful in elucidating the pathophysiology of SZ. Further, the EEG–GMV linkage was not abnormal in relatives of probands. Although the joint model (EEG + GMV) revealed a greater divergence compared to the individual models, it was statistically not significant. The biological and genetic architecture underlying the joint oscillatory and imaging abnormality can be probed to gain insights into the disease pathology and thereby find disease-specific mechanisms that can be investigated for treatment options. Future studies will involve integrating functional, task-specific imaging and genetic data with both EEG and brain morphometric changes that may aid in the development of biological based classification of psychotic illness to provide better treatment outcome.

## Author Contributions

CT, GP, JS and MK were responsible for study design and research proposal. All authors contributed to writing the manuscript. All authors read and corrected the manuscript. VC contributed to the methodology and analysis. BC contributed to the study design and interpretation. PS, BN, and SK managed the literature searches, performed the statistical data analyses, and wrote various sections of the final manuscript. All authors contributed to and have approved to the final manuscript.

## Conflict of Interest Statement

Dr. John Sweeney has received support from Takeda, BMS, Lilly, Roche, and Janssen. Dr. Matcheri Keshavan has received support from Sunovion. Dr. Carol Tamminga has received funding from Astellas, Eli Lilly, Intracellular Therapies, Lundbeck and Pure Tech Ventures. Other authors declare no financial interest in relation to the work described in this manuscript other than the grant funding.

## Footnotes

^1^http://adni.loni.usc.edu/

^2^http://dbm.neuro.uni-jena.de/vbm

^3^http://mialab.mrn.org/software
